# Whole genome sequence of multidrug-resistant *Staphylococcus haemolyticus* and *Enterococcus faecalis* isolates from public gymnasium equipment reveals evolving infection potential and resistance

**DOI:** 10.1371/journal.pone.0324894

**Published:** 2025-10-29

**Authors:** Goutam Banerjee, Pratik Banerjee

**Affiliations:** Department of Food Science and Human Nutrition, University of Illinois at Urbana-Champaign, Urbana, Illinois, United States of America; Hawassa University College of Medicine and Health Sciences, ETHIOPIA

## Abstract

Whole genome sequences (WGSs) of **Enterococcus fa*ecalis* S3 and *S*taphylococ*cus haemolyticus* S5, isolated from gymnasium equipment in Tennessee, USA, were analyzed. The genome sizes of *E. faecalis* S3 and *S. haemolyticus* S5 were approximately 3.0 Mb and 2.5 Mb, respectively. Both isolates were found to harbor genes conferring resistance to multiple antibiotics, including tetracycline, fluoroquinolone, and macrolide. Gene cluster analysis revealed a cyclic lactone inducer cluster in both strains, which is critical for quorum sensing-mediated pathogenicity. Multilocus sequence typing (MLST) identified *E. faecalis* S3 as ST40 and *S. haemolyticus* S5 as ST52. Notably, evolutionary analysis of gene contraction and expansion in these isolates revealed an expansion of genes associated with horizontal gene transfer. This expansion likely represents an evolutionary strategy to facilitate the spread of antibiotic resistance genes to other isolates. These findings offer valuable insights into the genomic apparatus responsible for antibiotic resistance and potential transmission mechanisms in human-associated environments.

## Introduction

Antimicrobial resistance (AMR) in human pathogens poses a significant global threat to human health. In 2019, bacterial AMR was directly linked to approximately 1.27 million deaths, with an estimated 4.95 million deaths attributed to AMR-related causes annually [1]. The persistence of AMR bacteria in the animal gut and different human-associated environments like soil, water, air, and environmental surfaces constitute a significant concern in the One Health program, which connects different sectors to provide better solutions for human, animal, and environmental health [2]. In this direction, Several international organizations, including the World Organization for Animal Health (OIE), the World Health Organization (WHO), and the Food and Agriculture Organization of the United Nations (FAO), have collaborated to develop the WHO Global Action Plan on Antimicrobial Resistance [3]. One of the challenges in managing infections caused by multidrug-resistant (MDR) bacteria is their inherent resistance to many antibiotics. Therefore, healthcare professionals face difficulties selecting effective antimicrobial therapies against illnesses associated with these bacteria. Among several MDR bacteria, Gram-positive bacteria such as *Enterococcus faecalis* [4] and *Staphylococcus haemolyticus* [5] are of particular concern in healthcare settings.

Coagulase-negative *S. haemolyticus* is prevalent in various natural environments, with a notable presence on human skin [6]. This bacterium poses a significant challenge in clinical settings owing to its high antibiotic resistance [7]. *S. haemolyticus* infections are commonly associated with sepsis in neonates, particularly in low-income countries [8]. Additionally, its ability to form biofilms on clinical instruments, especially catheters, contributes to a wide range of clinical manifestations, including prosthetic joint infections and bacteremia [7]. According to the Centers for Disease Control and Prevention (CDC), infections caused by drug-resistant *Staphylococcus* (mainly *S. aureus, S. epidermis*, and *S. haemolyticus*) bacteria result in approximately 323,700 cases annually in the USA, leading to 10,600 deaths [9].

Similarly, *E. faecalis* is commonly found in various environments, including soil, plants, water, animals, and the human gut [10,11]. In clinical settings, *E. faecalis* is recognized as a significant pathogen capable of causing a range of serious infections, including those in the urinary tract, bloodstream, surgical site, meningitis, and sepsis, which can pose substantial risks to human health [12,13]. According to data provided by the CDC, drug-resistant *Enterococcus* infections contribute to a considerable burden of disease in the United States, with an estimated 54,500 cases resulting in approximately 5,400 deaths annually [9].

The drug-resistant properties observed in different strains of *S. haemolyticus* and *E. faecalis* isolated from animal sources and hospital environments have been well-documented [11,14]. Collecting samples from diverse environmental settings such as water, soil, animal farms, indoor air, public restrooms, community activity centers, and fitness centers is gaining special attention within the One Health framework for antimicrobial surveillance [15]. The One Health approach integrates multidisciplinary efforts from environmental scientists, microbiologists, epidemiologists, and public health professionals to monitor and address the emergence of antimicrobial-resistant bacteria, their persistence in the environment, and their evolutionary adaptation [16]. Therefore, pathogen genomes obtained from systematic environmental sampling is essential to enrich the global database and support effective health management strategies.

The primary aim of this study is to perform a comprehensive genomic characterization of two multidrug-resistant bacterial strains, **S. haem*olyticus* S5 and *E. faecalis* S3, isolated from skin-contact surfaces of exercise equipment in a public fitness center. By analyzing their antibiotic resistance genes, virulence factors, and gene clusters, we seek to enhance understanding of the potential health risks posed by these bacteria in communal environments. Additionally, this study aims to contribute valuable genomic data to support surveillance efforts and inform public health strategies within the One Health framework.

## Materials and methods

### Sample collection

The bacterial isolates used in this study were collected as part of an environmental investigation, as described previously [17,18]. In brief, a total of 32 surface swab samples were collected (October 2013) from four fitness centers located in the Memphis metropolitan area, Tennessee, using cotton-tipped swabs (Sanicult™; Thermo Remel/Starplex Scientific Inc., Etobicoke, ON, Canada). Trained volunteers swabbed skin-contact surfaces of exercise equipment like Nautilus machines, power striders, stationary bikes, treadmills, elliptical machines, leg presses, dumbbells, stair handrails, and toilet handles. Swabs were immediately placed into tubes containing sterile diluent solution and transported in a refrigerated container to the laboratory for further analysis. Pure cultures of bacteria were isolated by repeated streaking on tryptic soy agar (TSA) medium.

### Bacterial culture and DNA extraction

Bacteria were cultured in tryptic soy broth (Sigma-Aldrich, USA) for 18 h at 37 °C, followed by DNA extraction using an Omega BIO-TEK bacterial DNA extraction kit (USA, Cat. No. D3350-02). The quality and quantity of the extracted DNA were assessed using a NanoDrop spectrophotometer (Thermo Scientific, USA) and 0.8% agarose gel, respectively.

### Library preparation, sequencing, and read quality assessment

The shotgun genomic library was prepared using an Illumina DNA preparation kit (Illumina cat no 20060060). The libraries were pooled and quantified in qPCR, followed by sequencing on a NovaSeq 6000 platform using V1.5 sequencing kit in paired-end mode (2 × 150 bp configuration). Fastq files, generated from the sequencing platform were used for downstream analysis. We used FastQC v0.11.9 to assess raw reads quality and fastp v0.23.4 [19] with parameters -f 14, -t 10, -F 14, -T 10, -q 25 for trimming.

### Genome assembly construction and de novo assembly statistics

We used a k-mer based de novo approach to assemble the genome fragments with SPAdes v3.15.5 [20] utilizing de Bruijn graph algorithms and the “--careful” option to generate longer contigs from short read data (2 × 150 bp). Assembly statistics were computed using QUAST v5.2.0 [21] with default parameters.

### Genome integrity evaluation and annotation prediction

The genome annotation was performed byProkka v1.14.6 [22] with specific settings: --kingdom bacteria, --addgenes, --evalue 1e-9, --rfam, -gcode 11, and –force. Following annotation, the completeness and contamination of the generated genomes were assessed using CheckM v1.2.2 [23].

### WGS-based prediction of putative antibiotic resistance genes (ARGs)

Putative antibiotic resistance genes in the two bacterial genomes were assayed in the RGI main server (https://card.mcmaster.ca/analyze/rgi) using the CARD (Comprehensive Antibiotic Resistance Database) database v3.2.1 [24] considering perfect (100% identity) and strict (>95% identity) parameters.

### WGS-based prediction of associated virulence factors

The genes associated with virulence determinants were predicted in abricate v1.0.1 (https://github.com/tseemann/abricate) using VFDB database [25]. We used the contig file generated from the assembled construction with 70% identity and 80% coverage parameters.

### Multilocus sequence typing (MLST) using WGS data

The sequence types (ST) of the two isolates were identified through multilocus sequence typing (MLST) analysis based on sequence variations in specific housekeeping genes. For this purpose, we used the mlst package v2.23.0, which facilitates MLST analysis, and accessed typing schemes from the PubMLST database [26].

### WGS-based phylogenetic tree construction

The phylogenetic relationship between the genomes of our isolates and other similar sequence type clones (*E. faecalis* ST40 and *S. haemolyticus* ST52) available in the NCBI Genome Database (https://www.ncbi.nlm.nih.gov/genome/) was analyzed using an online tool named REALPHY (https://realphy.unibas.ch/realphy/), which integrates Bowtie2 for mapping-based alignment and PhyML for inferring phylogenetic trees. For this purpose, five genomes of *E. faecalis* ST40 and five genomes of *S. haemolyticus* ST52 were downloaded to construct phylogenetic trees, allowing a comparative assessment of genetic relatedness. Detailed information about these isolates, including accession numbers and metadata, is provided in S1 Table. This analysis helped contextualize the genomic characteristics of our isolates within the broader phylogenetic landscape of these sequence types.

### Comparative proteomics and gene family expansion/contraction analysis

To elucidate the evolutionary relationships between **S. haemo*lyticus* S5, *E. faecalis* S3, and other closely related strains within their respective species, we utilized the OrthoFinder algorithm as implemented in the OrthoVenn 3 package [27]. This analysis was conducted with an e-value threshold of 1e − 5 and an inflation value set to 1.5, enabling accurate identification and clustering of orthologous gene families. To further explore the evolutionary dynamics, we performed gene family expansion and contraction analysis using CAFÉ v5.0 [28]. This program employs a birth-and-death process model to estimate rates of gene gain and loss across a given phylogenetic tree. By analyzing the distribution of family sizes under this model, we assessed the significance of observed differences in gene family sizes among taxa, providing valuable insights into the adaptive evolution of these strains.

### Gene cluster prediction

To identify potential gene clusters within the genomes of **S. haemol*yticus* S5 and *E. faecalis* S3, we utilized the bacterial version of the antiSMASH v6.0 tool [29]. This powerful bioinformatics tool specializes in the prediction of secondary metabolite biosynthetic gene clusters. The nucleotide sequences and corresponding GenBank files generated during the genome annotation process were used as inputs. Gene cluster prediction was performed using the ‘strict’ detection parameter to ensure high-confidence results. This approach allowed for the identification of genomic regions potentially involved in the production of bioactive compounds, providing further insights into the functional and adaptive capabilities of these isolates.

## Results and discussion

### Raw sequence read, quality check, and trimming

The sequencing platform generated 1,063,199 and 901,900 paired-end reads for **E. f*aecalis* S3 and *S. haemolyticus* S5, respectively (S2 Table). The initial quality assessment revealed Q30 bases of 87.86% and 86.96% for **E. fa*ecalis* S3 and *S. haemolyticus* S5, respectively, prior to filtering. Subsequently, theraw reads were trimmed to improve read quality for downstream analysis, resulting in increased Q30 bases to 88.28% for *E. faecalis* S3 and 87.29% for *S. haemolyticus* S5 (S2 Table). The high percentage of Q30 bases indicates the overall good quality of the sequencing data.

### Genome assembly from fragmented DNA sequence and annotation

The trimmed reads were used for genome assembly, resulting in 39 contigs for *E. faecalis* S3, with the largest contig spanning 590,494 bp and an N50 value of 183,877 bp. For *S. haemolyticus* S5, the assembly produced 58 contigs, with the largest contig measuring 249,943 bp and an N50 value of 93,523 bp. Assembling complete genomes from fragmented data is challenging and critical in whole-genome sequence (WGS) analysis. Various assemblers are available, but we opted for the k-mer based *de novo* assembler SPAdes [30] based on the standard recommendations [31], followed by annotation using Prokka. Annotation revealed GC contents of 37.41% for **E. fa*ecalis* S3 and 32.77% for *S. haemolyticus* S5. *E*. faecali*s* S3 had 2826 CDS, 5 rRNA, 78 misc_RNA, and 52 tRNA genes, while **S. haemo*lyticus* S5 had 2385 CDS, 12 rRNA, 78 misc_RNA, and 64 tRNA genes. Completeness and contamination assessments showed high completeness scores of 99.63% for **E. faec*alis* S3 and 99.62% for *S. haemolyticus* S5, with no detectable contamination, indicating that the genomes are nearly complete and suitable for further genomic analysis.

### WGS-based antimicrobial resistance gene prediction

The putative antimicrobial resistance (AMR) genes were predicted and are presented in Table 1. **E. faec*alis* S3 harbored multiple AMR genes, including *dfr(E), tet(M), efrA, efrB, IsaA, IreK*, and *emeA*, conferring resistance to various antibiotic classes such as diaminopyrimidine, tetracycline, macrolide, streptogramin, and pleuromutilin (Table 1). Additionally, the *emeA* gene in *E. faecalis* encodes an efflux pump that expels biocides and ammonium-containing compounds commonly used in disinfectants [32]. A previous study reported a high prevalence of *emeA* gene (42.8%) among clinical isolates of *E. faecalis* [32]. The presence of the *emeA* gene in **E.* faecalis* S3 is prevalent, and therefore, cleaning surfaces with ammonium-based disinfectants alone may not effectively eliminate this pathogen. However, phenotypic resistance was not assessed in this study. Previous studies have also identified several AMR genes, such *as lsa(A), RlmA(II), LiaR, LiaS, dfr(E)*, and *tet(M)*, in *E. faecalis* isolated from diseased fish [22] and resistance to chloramphenicol, linezolid, and teicoplanin in dental plaque isolates [33]. In contrast, **S. haemolyt*icus* S5 exhibited resistance to macrolide (*mphC*), aminoglycoside (*ANT(4’)-Ia*), tetracycline (*tetK*), and fluoroquinolone (*qacA*) antibiotics (Table 1). A study of 365 *S. haemolyticus* isolates found 91.3% and 85.4% resistance to cefoxitin and erythromycin, respectively, and 57.3%, 52.8%, and 3.7% resistance to co-trimoxazole, clindamycin, and linezolid, respectively [34]. The presence of multidrug-resistant (MDR) bacteria on environmental surfaces, such as those found in healthcare facilities [35], public restrooms [36], supermarket shopping carts [37], and fitness facilities [38], is a growing concern for community health. Surveillance of these environments is a key component of the One Health approach. This approach recognizes that human health is closely linked to the surrounding environment, making regular surveillance essential for effective public health management [39]. Through source tracking, monitoring, prevention strategies, and intervention policies, the One Health framework aims to control the spread of MDR bacteria and protect community health. In April 2019, the Interagency Coordination Group on Antimicrobial Resistance, comprising the WHO, OIE, and FAO, presented a report titled “*We Can’t Wait: Securing the Future Against Drug-Resistant Infections*” to the UN Secretary-General. To combat this situation, all agencies agreed to raise awareness of the AMR problem among general people, healthcare professionals, and policymakers to control the spread of antibiotic use and AMR bacteria [40].

**Table 1:** Putative antibiotic resistance genes present in **E. faeca*lis* S3 and *S. haemolyticus* S5.

Bacterial isolates	AMR genes	AMR Gene Family	Drug Class	Resistance Mechanism	% nucleotide Identity of Matching Region
*E. faecalis* S3	*dfr(E)*	trimethoprim-resistant dihydrofolate reductase	diaminopyrimidine	antibiotic target replacement	97.56
	*tet(M)*	tetracycline-resistant ribosomal protection protein	tetracycline	antibiotic target protection	98.75
	*efrA*	ATP-binding cassette (ABC) antibiotic efflux pump	macrolide, fluoroquinolone, rifamycin	antibiotic efflux	99.65
	*efrB*	ATP-binding cassette (ABC) antibiotic efflux pump	macrolide, fluoroquinolone, rifamycin	antibiotic efflux	97.58
	*IreK*	Serine/threonine kinases	cephalosporin	reduced permeability to antibiotic	98.89
	*lsaA*	Lsa-type ABC-F protein	lincosamide, streptogramin, streptogramin A, streptogramin B, pleuromutilin	antibiotic target protection	99.6
	*emeA*	multidrug and toxic compound extrusion (MATE) transporter	disinfecting agents and antiseptics	antibiotic efflux	97.67
*S. haemolyticus* S5	*tet(K)*	major facilitator superfamily (MFS) antibiotic efflux pump	tetracycline	antibiotic efflux	100
	*msrA*	msr-type ABC-F protein	macrolide, streptogramin, streptogramin B	antibiotic target protection	98.57
	*mphC*	macrolide phosphotransferase	macrolide	antibiotic inactivation	98.66
	*qacA*	major facilitator superfamily (MFS) antibiotic efflux pump	fluoroquinolone	antibiotic efflux	99.81
	*ANT(4’)-Ia*	ANT(4’)	aminoglycoside	antibiotic inactivation	99.21

Public gymnasium centers are high-contact environments where individuals frequently share fitness equipment and floor surface, often under conditions of limited or inconsistent disinfection. The combination of perspiration-induced warmth and moisture, along with frequent skin-to-skin contact, creates an optimal environment for bacterial persistence and transmission [41]. The detection of MDR bacteria in such settings is critical for understanding potential non-clinical transmission routes and for guiding effective public health interventions.

### WGS-based virulence factor prediction

The prediction of virulence factors is crucial for assessing the pathogenic potential of a pathogen. In our analysis, **E. fa*ecalis* S3 harbored 19 virulence genes (identity 92–99%, coverage 86–100%), including biofilm-associated pilus genes (*EbpA, EbpB, EbpC*), gelatinase (*gelE*), and serine protease (*sprE*), known for enhancing pathogenicity (Table 2). Previous studies have highlighted the importance of the *ebpABC-bps* gene cluster in attachment, a critical step in biofilm formation initiation [42]. Furthermore, gelatinase and serine protease encoded by *gelE* and *sprE* are also reported as major virulence factors in *E. faecalis* [43]. In comparison, **S. haemolyti*cus* S5 showed only two virulence genes, *ClpP* and *ClpC*, with *ClpP* known for enhancing infectivity in *Staphylococcus* species [44]. Thus, the presence of virulence genes alongside antibiotic resistance properties suggests that these isolates may pose a higher risk of pathogenicity.

**Table 2:** Predicted virulence factors in **E. faeca*lis* S3 and *S. haemolyticus* S5.

Bacterial isolate	Genes	Coverage (%)	Identity (%)	Function
*E. faecalis* S3	*bopD*	100	99.11	Sugar-sensing transcriptional regulator
	*srtC*	100	99.18	Pilus associated sortase
	*ebpC*	100	99.05	Endocarditis and biofilm-associated
	*ebpB*	100	99.37	Endocarditis and biofilm-associated
	*ebpA*	100	99.43	Endocarditis and biofilm-associated
	*asa1*	100	96.3	Aggregation substance for conjugation
	*sprE*	100	98.71	Serine protease
	*gelE*	100	98.76	Gelatinase production
	*fsrC*	100	99.55	Histidine kinase putative (quorum sensing)
	*fsrB*	100	98.77	agrBfs protein (quorum sensing)
	*fsrA*	100	98.63	Response regulator (quorum sensing)
	*EF0818*	100	99.2	Polysaccharide lyase family 8
	*EF3023*	100	98.54	polysaccharide lyase family 8
	*cpsB*	100	99.5	Phosphatidate cytidylyltransferase
	*cpsA*	100	99.63	Undecaprenyl diphosphate synthase
	*fss2*	86.74	92.5	Fibrinogen binding protein
	*ace*	100	96.99	Collagen adhesin protein
	*efaA*	100	99.57	Endocarditis specific antigen
	*fss1*	100	98.66	Surface protein Fss1
*S. haemolyticus* S5	*clpC*	91.43	70.59	Regulate cap operon related virulence
	*clpP*	95.31	76.98	Global regulator of virulence related genes

### Multilocus sequence typing (MLST) of the isolated strains using WGS

**S. haemoly*ticus* S5 was identified as ST52 based on allelic variations in arcC(2), SH_1200(1), hemH(6), leuB(1), SH1431(2), cfxE(1), and Ribose_ABC(4). The prevalence of sequence type (ST) 52 is relatively uncommon in nature. However, a previous study reported the emergence of ST52 strains of *S. haemolyticus* isolated from hospitals and hospital-associated environments in China [14]. Interestingly, all these isolates exhibited resistance to a broad spectrum of antibiotics. In contrast, **E. faec*alis* S3 was assigned ST40 based on allelic variations in gdh(3), gyd(6), pstS(23), gki(12), aroE(9), xpt(10), and yqiL(7). ST40 is a major clone reported across diverse habitats, including humans, animals, and environmental sources [45] and is associated with infections [46]. MLST is a standardized, sequence-based method that characterizes bacterial strains by analyzing the allelic profiles of several housekeeping genes [47]. This makes it a valuable tool for epidemiological tracking, antimicrobial resistance monitoring, and understanding the population structure and evolutionary history of pathogens [48]. A comparative genomics analysis revealed that the distribution of virulence-associated genes was not significantly different between *E. faecalis* ST40 strains isolated from animal and human sources, nor between those from commensal and clinical backgrounds [45]. In contrast, *S. haemolyticus* ST52 is an uncommon strain type and has primarily been reported from home environments [49]. Its detection in a public gymnasium setting may indicate potential environmental dissemination and warrants further investigation into its reservoirs, transmission pathways, and public health implications. Furthermore, pathogenic strains with an MLST designation can be compared with the same ST isolates in the global database to understand genome-relatedness and antibiotic resistance patterns. Thus, MLST can guide antibiotic selection against AMR bacteria, though correlations between MLST and resistance can vary by species, as seen in recent studies with *Staphylococcus epidermidis* [50].

### WGS-based phylogenetic tree construction of the sequenced strains

**Fig 1:**
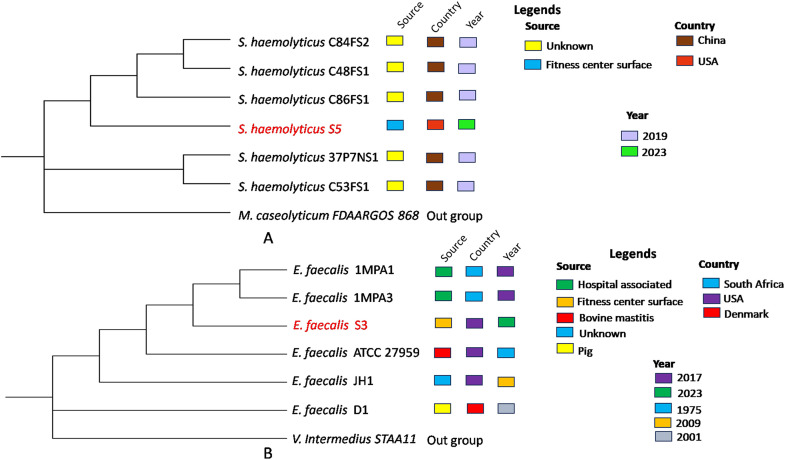
Core genome-based phylogenetic tree of the selected strains. (A) and (B) indicate the phylogenetic relationship between **S. haemolyti*cus* S5 and *E. faecalis* S3 with other strains isolated from different surfaces. *Macrococcoides caseolyticum* FDAARGOS 868 and *Vagococcus intermedius* STAA11 were taken as outgroups for the construction of a phylogenetic tree for *S. haemolyticus* S5 and *E. faecalis* S3, respectively.

A whole-genome sequencing (WGS)-based phylogenetic tree was constructed using five strains of *S. haemolyticus* ST52 and five strains of *E. faecalis* ST40 (S1 Table). The analysis revealed that **S. haemolyt*icus* S5 exhibited a close genetic relationship with *S. haemolyticus* C86FS1, a strain isolated from hospital-associated feces in China (Fig. 1A). Notably, like **S. haemoly*ticus* S5, the *S. haemolyticus* C86FS1 strain was resistant to multiple antibiotics, including ciprofloxacin, gentamicin, and linezolid [14]. Similarly, **E. faeca*lis* S3 showed a close relationship with *E. faecalis* 1MPA3, a strain isolated from a hospital-associated environment in South Africa (Fig. 1B). This genetic proximity highlights the global dissemination of specific antibiotic-resistant clones in healthcare-associated environments.

### Protein ortholog and gene family expansion and contraction

**Fig 2:**
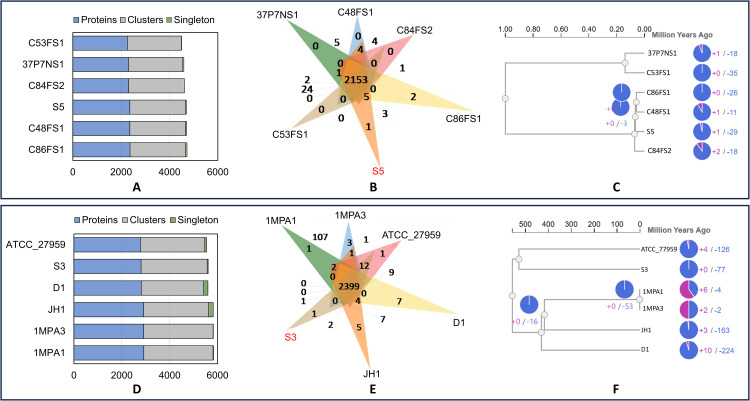
The protein cluster and protein ortholog comparison. **(A)** An overview of the protein clusters and singletons in **E. faeca*lis* S3 and other closely related strains, **(B)** The distribution of shared and unique protein clusters among different strains of *E. faecalis*, **(C)** The expansion and contraction of gene families in **E. faec*alis* S3 in comparison with other strains. **(D)** The protein clusters in **S. haemolyticu*s* S5 and its closely related strains, **(E)** The number of shared and unique protein clusters in **S. haemolytic*us* S3 in comparison to other strains. **(F)** The evolutionary tree indicates the number of gene family expansions and contractions in **S. haemolyticu*s* S5 over the evolutionary period.

We compared protein orthologs between **S.haemoly*ticus* S5 and five other strains belonging to the ST52 clone (S1 Table). The analysis identified 2,359 proteins in 2,302 clusters and 49 singletons in **S. haemoly*ticus* S5 (Fig 2A). Among these, 2,153 clusters were shared with other strains, while **S. haemolyti*cus* S5 exhibited one unique cluster (Fig 2B). Gene family expansion and contraction analysis revealed that **S. haemolyti*cus* S5 had one expanded gene family (GO:0032196), while 29 gene families, primarily associated with metabolic processes (S3 Table), were contracted in this strain (Fig 2C). The expanded gene family encodes a protein, the putative transposon Tn552 DNA-invertase Bin3, which is a type of mobile genetic element. This protein plays a critical role in the horizontal transfer of antibiotic resistance genes, contributing to the dissemination of resistance within microbial communities [51]. The expansion of this gene family in **S. haem*olyticus* S5 underscores a potential emerging threat, as it enhances the strain’s ability to spread antibiotic resistance within the community.

The protein orthology analysis among *E. faecalis* strains (S1 Table) revealed that **E. fae*calis* S3 possesses 2,807 proteins organized into 2,757 clusters and 42 singletons (Fig 2D). Comparative orthology indicated that **E. fae*calis* S3 contains a unique cluster not found in the other strains (Fig 2E). Evolutionary analysis of **E. faeca*lis* S3 identified 77 contracted gene families, but no gene family expansions were observed (Fig 2F). These contracted gene families are associated with diverse biological functions, including metabolic processes, carbohydrate transport, responses to stimuli, and other critical pathways (S4 Table). The lack of gene family expansion in **E. fa*ecalis* S3, coupled with significant contractions in key functional categories, may influence its adaptability and pathogenic potential in specific environments. These findings provide valuable insights into the evolutionary dynamics of **E. f*aecalis* S3 and its unique functional attributes.

### WGS-based gene cluster prediction

**Fig 3:**
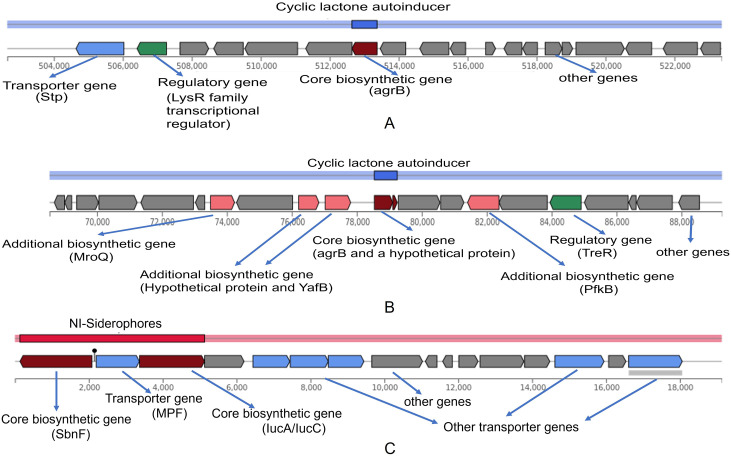
The predicted gene cluster in both *E. faecalis* S3 and *S. haemolyticus* S5. (A) The cyclic lactone autoinducer gene cluster in *E. faecalis* S3. The core biosynthetic gene *agrB* encodes regulator protein B, (B) and (C) represent cyclic lactone autoinducer and NI-siderophore gene clusters predicted in *S. haemolyticus* S5, respectively. Both gene clusters are involved in pathogenicity.

The gene cluster prediction analysis is depicted in Fig. 3. It reveals the presence of a cyclic lactone autoinducer gene cluster in **E. faeca*lis* S3, consisting of a biosynthetic gene (*agrA*), a regulatory gene (hypothetical protein), and a transporter gene (*stp*) (Fig. 3A). However, a protein BLAST search unveiled that the regulatory hypothetical protein is encoded by a transcriptional family gene *lysR*, exhibiting 100% similarity with *E. faecalis* strain ADL-337. The agr system, involving agrB, regulates quorum-sensing responses and virulence in *E. faecalis* and other Gram-positive pathogens [52]. In **S. haemolyti*cus* S5, two gene clusters were identified: cyclic lactone autoinducer and NI-Siderophores (Fig 3B). The lactone-containing autoinducer peptide activates the agr system, a global regulator in Staphylococci that influences virulence-associated protein production [53]. The NI-Siderophores cluster includes *SnbF*, which is involved in siderophore production, which is crucial for iron acquisition and enhances virulence in *Staphylococcus* [54]. These gene clusters highlight the pathogenic potential of **E. fae*calis* S3 and *S. haemolyticus* S5, underscoring their ability to regulate virulence and adapt to host environments.

## Conclusions

The presence of MDR bacteria in public spaces [55–57] is a serious health concern. This study focused on the persistence of two multidrug-resistant bacterial isolates, **E. faec*alis* S3 and **S. haemolyt*icus* S5, collected from public gymnasium equipment. From a One Health perspective, the availability of MDR bacterial genomes can enhance surveillance and health management efforts. Using advanced genome assembly methods, we obtained near-complete sequences (>99.5% completeness), providing detailed insights into the genetic makeup of these pathogens. Both isolates carried multiple antibiotic resistance genes, indicating their potential to resist commonly used antimicrobials; however, phenotypic antibiotic resistance was not assessed. Additionally, several virulence factors were identified, including biofilm-associated genes in **E. f*aecalis* S3, suggesting enhanced persistence in public environments. These findings highlight the potential health risks posed by MDR bacteria in communal spaces. Although this study is limited by reliance on genome-based bioinformatics analysis without phenotypic antibiotic resistance data, we recommend using hand sanitizer after using public gymnasium equipment to minimize the contamination risk.

## Supporting information

S1 Table Reference strains of *Enterococcus faecalis* and *Staphylococcus haemolyticus* downloaded from NCBI Genome Database. (DOCX)

S2 Table Reads statistics before and after processing. (DOCX)

S3 Table The contracted gene family associated GO and their function in *S. haemolyticus* S5. (DOCX)

S4 Table The contracted gene family associated GO and their function in *E. faecalis* S3. (DOCX)
