# Supplementary material for: Whole genome sequence of multidrug-resistant Staphylococcus haemolyticus and Enterococcus faecalis isolates from public gymnasium equipment reveals evolving infection potential and resistance
Source: PLoS One. 2025 Oct 29;20(10):e0324894. doi: 10.1371/journal.pone.0324894 (PMC12571285; doi:10.1371/journal.pone.0324894)
Supplement: S1 Table — (DOCX) [file pone.0324894.s001.docx]

**S1 Table.** **Reference strains of *Enterococcus faecalis* and *Staphylococcus haemolyticus* downloaded from NCBI Genome Database.**

| **Reference strains** | **Strains** | **Isolation sources** | **Countries** | **RefSeq. Identifier** |
| --- | --- | --- | --- | --- |
| *Enterococcus faecalis* | ATCC 27959  D1  1MPA1  1MPA3  JH1 | Bovine mastitis  Pig  Hospital environment  Hospital environment  Unknown | USA  Denmark  South Africa  South Africa  USA | GCF_000395265.1  GCF_000393575.1  GCF_017642245.1  GCF_017642265.1  GCA_000157375.1 |
| *Staphylococcus*  *haemolyticus* | C86FS1  C48FS1  C84FS2  C53FS1  37P7NS1 | Unknown  Unknown  Unknown  Unknown  Unknown | China  China  China  China  China | GCF_022486125.1  GCF_022486065.1  GCF_022485685.1  GCF_022486265.1  GCF_022486415.1 |
