# Supplementary material for: Whole genome sequence of multidrug-resistant Staphylococcus haemolyticus and Enterococcus faecalis isolates from public gymnasium equipment reveals evolving infection potential and resistance
Source: PLoS One. 2025 Oct 29;20(10):e0324894. doi: 10.1371/journal.pone.0324894 (PMC12571285; doi:10.1371/journal.pone.0324894)
Supplement: S2 Table — (DOCX) [file pone.0324894.s002.docx]

**S2 Table.** **Reads statistics before and after processing.**

| **Bacterial isolate** | **Raw reads** | | | **Processed reads** | | |
| --- | --- | --- | --- | --- | --- | --- |
| *E. faecalis* S3 | Read count | Q20% | Q30% | Read count | Q20% | Q30% |
|  | 2.12 M | 95.10 | 87.86 | 2.10 M | 95.41 | 88.28 |
| *S. haemolyticus* S5 | 1.80 M | 94.81 | 86.96 | 1.78 M | 95.08 | 87.29 |
