# Supplementary material for: Whole genome sequence of multidrug-resistant Staphylococcus haemolyticus and Enterococcus faecalis isolates from public gymnasium equipment reveals evolving infection potential and resistance
Source: PLoS One. 2025 Oct 29;20(10):e0324894. doi: 10.1371/journal.pone.0324894 (PMC12571285; doi:10.1371/journal.pone.0324894)
Supplement: S3 Table — (DOCX) [file pone.0324894.s003.docx]

**S3 Table. The contracted gene family associated GO and their function in *S. haemolyticus* S5.**

| **Slimmed GO** | **Biological process** | **Unique gene count** |
| --- | --- | --- |
| GO:0006139 | Nucleobase-containing compound metabolic process | 3 |
| GO:0006725 | Cellular aromatic compound metabolic process | 3 |
| GO:0006793 | Phosphorus metabolic process | 2 |
| GO:0006807 | Nitrogen compound metabolic process | 3 |
| GO:0006811 | Ion transport | 1 |
| GO:0008150 | Biological_process | 4 |
| GO:0008152 | Metabolic process | 3 |
| GO:0009117 | Nucleotide metabolic process | 2 |
| GO:0009987 | Cellular process | 3 |
| GO:0016070 | RNA metabolic process | 1 |
| GO:0032196 | Transposition | 3 |
| GO:0043170 | Macromolecule metabolic process | 1 |
| GO:0044237 | Cellular metabolic process | 3 |
| GO:0044238 | Primary metabolic process | 3 |
| GO:0046483 | Heterocycle metabolic process | 3 |
| GO:0050896 | Response to stimulus | 1 |
| GO:0065007 | Biological regulation | 3 |
