# Supplementary material for: Whole genome sequence of multidrug-resistant Staphylococcus haemolyticus and Enterococcus faecalis isolates from public gymnasium equipment reveals evolving infection potential and resistance
Source: PLoS One. 2025 Oct 29;20(10):e0324894. doi: 10.1371/journal.pone.0324894 (PMC12571285; doi:10.1371/journal.pone.0324894)
Supplement: S4 Table — (DOCX) [file pone.0324894.s004.docx]

**S4 Table.** **The contracted gene family associated GO and their function in *E. faecalis* S3**

| **Slimmed GO** | **Biological process** | **Unique gene count** |
| --- | --- | --- |
| GO:0006082 | Organic acid metabolic process | 1 |
| GO:0006139 | Nucleobase-containing compound metabolic process | 4 |
| GO:0006259 | DNA metabolic process | 2 |
| GO:0006260 | DNA replication | 1 |
| GO:0006304 | DNA modification | 1 |
| GO:0006412 | Translation | 1 |
| GO:0006725 | Cellular aromatic compound metabolic process | 4 |
| GO:0006807 | Nitrogen compound metabolic process | 4 |
| GO:0006810 | Transport | 2 |
| GO:0006811 | Ion transport | 1 |
| GO:0008150 | Biological_process | 10 |
| GO:0008152 | Metabolic process | 6 |
| GO:0008643 | Carbohydrate transport | 2 |
| GO:0009987 | Cellular process | 2 |
| GO:0016070 | RNA metabolic process | 2 |
| GO:0032196 | Transposition | 5 |
| GO:0032502 | Developmental process | 1 |
| GO:0043170 | Macromolecule metabolic process | 4 |
| GO:0044237 | Cellular metabolic process | 5 |
| GO:0044238 | Primary metabolic process | 4 |
| GO:0046483 | Heterocycle metabolic process | 4 |
| GO:0050896 | Response to stimulus | 2 |
| GO:0051179 | Localization | 2 |
| GO:0051234 | Establishment of localization | 2 |
| GO:0051704 | Multi-organism process | 3 |
| GO:0065007 | Biological regulation | 1 |
